# *Bacillus thuringiensis* and insects: a century of intimate history

**DOI:** 10.1128/jb.00381-25

**Published:** 2026-02-17

**Authors:** Leyla Slamti, Didier Lereclus

**Affiliations:** 1 Micalis Institute, INRAE, AgroParisTech, Université Paris-Saclay, Jouy-en-Josas, France; Dartmouth College Geisel School of Medicine, Hanover, New Hampshire, USA

**Keywords:** *Bacillus anthracis*, *Bacillus cereus*, biopesticide, Bt plant, gene expression, infection, insect, pathogenicity, plasmid, quorum sensing, toxin, transposon

## Abstract

Within the vast *Bacillus cereus* group, two bacterial species have stood out for over a century: *Bacillus anthracis* for its pathogenicity to mammals, and *Bacillus thuringiensis* for its remarkable and economically exploitable activity against invertebrates. One hundred years of extensive research around the world have unraveled the sophisticated mechanisms that make *B. thuringiensis* a formidable weapon designed to kill insects, exploiting them as an ecological niche for its proliferation. Evolution has led to the selection of a great diversity of highly specific toxins targeting a wide range of insects and nematodes. Bacteria have developed transcriptional, post-transcriptional, and post-translational mechanisms that enable the massive production of these toxins as crystalline inclusions. Virulence and adaptation factors, together with regulation systems, have also been selected to enable the bacterium to make the most of the ecological niche provided by insects. In addition to their interest in the bacterium, the biological tools and processes developed by *B. thuringiensis* can be exploited by mankind to create insect-resistant plants, overproduce proteins, crystallize them, and gain a better understanding of the microbial world. All the research carried out on *B. thuringiensis* over the last century has made this bacterium a remarkable study model and biotechnological resource, revealing all the subtlety and power of the mechanisms that a microorganism has been able to acquire in the course of its evolution.

## INTRODUCTION

The *Bacillus cereus* group contains a large variety of sporulating gram-positive bacteria distributed in several species, the best known being *Bacillus thuringiensis*, *Bacillus cereus,* and *Bacillus anthracis*, frequently designated as “the Good, the Bad, and the Ugly,” respectively (1). These closely related Bacilli are able to colonize hosts as diverse as insects and mammals, or food (2). The “Good” species, *B. thuringiensis* (also known as Bt), is composed of bacterial strains producing protein toxins forming a crystal with insecticidal or nematicidal properties (3, 4). Some have been used for decades as biopesticides against insect pests in agriculture and forestry, while others are used to control black flies and mosquitoes. In addition, since the end of the last century, several Bt toxins have been widely used to construct genetically modified plants resistant to insect pests (5). Due to its interest in agriculture and human health, and consequently to its high economic potential, Bt has generated a considerable number of basic and applied scientific studies over much of the 20th century up to the present day. Altogether, the research on Bt has provided a wealth of original information in various fields of biology and biochemistry, such as protein crystallization, mode of action of toxins, plasmids, transposons, regulation of gene expression, bacterial-host interactions, etc. Based on its exceptional properties, Bt can be regarded as a model bacterium for several aspects, including the diversity of virulence and toxin genes, the capacity to produce a crystal inclusion, and the network of quorum-sensing (QS) systems orchestrating virulence, necrotrophism, and sporulation. The designation “Bad bacteria” can be assigned to the species *B. cereus stricto sensu*, whose members are frequently associated with food-borne intoxications (2), although some strains can be used as probiotics for plants and animals. Of course, as the etiological agent of anthrax, *B. anthracis* is the “Ugly” member of the *B. cereus* family (2). Part of the data acquired from Bt can be applied to *B. cereus sensu stricto* and, to a lesser extent, to *B. anthracis*. We attempt here to describe the major milestones that paved the way for a century of research and made Bt an organism suited to study a bacterial species (or group) with respect to its ecological niche.

## THE ORIGINS

The three main members of the *B. cereus* group were discovered over a century ago, making a major contribution to the extraordinary development of microbiology starting in the second half of the 19th century. This remarkable breakthrough in biological sciences was initiated by the revolutionary discoveries of Agostino Bassi, Louis Pasteur, and Robert Koch, showing that microorganisms were the cause of various human and animal diseases. As early as 1836, Bassi demonstrated that a devastating silkworm disease (known as muscardine) was transmitted by a fungus, now known as *Beauveria bassiana* (6). In 1870, by studying other outbreaks in silkworm farms of southern France, Louis Pasteur clearly demonstrated that the disease known as flacherie was contagious and that this contagion was due to small corpuscles called “vibrions” (7). Viewed through 21st-century eyes, the micrographs provided by Pasteur strongly suggest that the “vibrions” found in the guts of contaminated *Bombyx mori* larvae are spore-forming bacteria that look suspiciously like bacilli (https://gallica.bnf.fr/ark:/12148/btv1b8626103j/f303.item). However, the absence of crystal inclusions in the sporulating bacterial cells does not allow us to postulate that they were Bt.

A few years after the studies on silkworm, the harsh but productive competition between Pasteur and Koch led to establishing the causal role of a specific bacterium in the etiology of anthrax (8, 9). Based on Ferdinand Cohn’s work on bacteria and the characterization of *B. anthracis* (10), Robert Koch demonstrated for the first time that a bacterium, *B. anthracis*, was directly responsible for the transmission of a disease, anthrax.

Bt was isolated 30 years later, in 1901, from infected silkworm larvae responsible for a severe flacherie in Japan and clearly identified as the causal agent of the disease, which was a major scourge for the silk industry (11). The bacterium was definitively characterized in 1915 by Ernst Berliner, who isolated it from the cadavers of flour moth larvae collected in a flour mill in Thuringia (Germany), giving it the name *Bacillus thuringiensis* (12).

Unlike Bt and *B. anthracis*, the first isolation of *B. cereus sensu stricto* was not from contaminated animals. It was carried out in the environment of a cowshed, and the bacterium was characterized as a spore-forming bacterium with the distinctive ability to cause rapid liquefaction of gelatin (13). It was only in the second half of the 20th century that some *B. cereus* strains were associated with illnesses, such as gastrointestinal diseases causing diarrheal or emetic syndromes, or with opportunistic infections such as endophthalmitis (14–16).

If we now focus our attention on Bt, it has been clearly established since the 1950s that this bacterium has a significant pathogenic potential toward various lepidopteran insects (17), and histopathological analyses revealed that, after ingestion of the bacteria, the infection consisted of rapid toxemia causing gut paralysis, followed by slow septicemia leading to the death of the larvae (18–20). At roughly the same time, three major discoveries complementing these initial data were made by Canadian scientists. The first was to show that the spores were accompanied by a large inclusion in the form of a diamond-shaped (or bi-pyramidal) crystal (21); the second was to demonstrate that the insect paralysis resulted from ingestion of these parasporal crystalline inclusions (22); and finally, it was shown that the crystals consisted of proteins (23) originally called ∂-endotoxins (24) and designated today as Cry and Cyt proteins (25–27). Following these initial fundamental results, it was shown that, once ingested by the larvae, the crystals dissolved in the alkaline environment of the gut, and the ∂-endotoxins were cleaved into active toxins by gut proteases (28).

In parallel with these basic studies, several field trials carried out in different parts of the world have demonstrated the efficacy of Bt against certain lepidopteran crop pests (17)—and in 1960, the Institut Pasteur (Paris, France) filed the first patent on Bt, describing a process for obtaining biological products to control insect pests in agriculture. As a result, the economic interest generated by Bt in the 1960s led to significant academic and industrial research. This interest increased sharply in 1977 with the discovery of the Bt strain *israelensis* active against mosquitoes (29), then in 1983 with the isolation of the strain *tenebrionis* active against Coleoptera (30). By showing that the activity of Bt was not restricted to lepidopteran insects, these results opened up major economic prospects for both crop protection and vector control.

## BT: A MODEL BACTERIUM

Over the last 40 years, Bt has proven to be an outstanding bacterial model in four main fundamental aspects, all showing the remarkable capacity of adaptation of this bacterium: (i) plasmids and mobile genetic elements, (ii) diversity of insecticidal toxins, (iii) regulation of ∂-endotoxin gene expression, and (iv) pathogenic lifestyle. In addition, Bt has had—and continues to have—a great impact on the control of parasitic disease vectors and on crop protection as a biopesticide, but also as a prolific supplier of insecticidal toxin genes for transgenic plants.

### Plasmids and mobile genetic elements

Bacteria of the *B. cereus* group, and particularly those of the Bt species, harbor a large number of plasmids, including linear plasmids (tectiviruses), small rolling-circle plasmids, and large theta-type plasmids, which, as a whole, may represent up to 11% of the total genomic DNA (31). Except for functions related to replication, mobilization, or segregational stability, the small plasmids are generally regarded as cryptic, with their main interest being, for molecular biologists, to serve as cloning vector (32–36). From this point of view, a remarkable Bt plasmid, selected for its stability properties, has been used to develop cloning and expression vectors (the pHT plasmid series), which are widely used in Bacilli and other gram-positive bacteria like *Listeria* (37, 38). The term *cryptic* largely reflects the fact that the functions of these small plasmids are not characterized. However, an 8.5-kb plasmid (pHT-8.1) from a Bt *kurstaki* strain has been shown to carry *rap-phr* genes, which encode a quorum-sensing system involved in the Spo0A phosphorelay that regulates sporulation (39). Interestingly, this Rap-Phr plasmid system exerts its activity specifically in infected insect larvae, indicating that small plasmids might contribute to the adaptive properties of Bt by influencing the development of bacteria under particular conditions, notably in their ecological niche. Besides this example provided by a Bt strain, similar Rap-Phr systems involved in the sporulation process are most often found on larger plasmids such as pXO1 in *B. anthracis*, pCER270 in the emetic strains of *B. cereus,* or the conjugative plasmid pAW63 in Bt (40–43).

While the small plasmids are generally cryptic, some large plasmids (> 50 kb) play a major role in determining the specific pathogenicity of the bacteria (2). In *B. anthracis*, all the genes responsible for anthrax are located on two large plasmids, pXO1 and pXO2 (44, 45); in the *B. cereus* emetic strain, the genes involved in the synthesis of the toxin cereulide are located on the plasmid pCER270 (46, 47); and in all Bt strains, the genes encoding the insecticidal toxins (Cry, Cyt, or Vip3) are on large plasmids (3, 48, 49). These plasmids may also harbor genes encoding specific transcriptional regulators involved in the expression of the toxin genes, as is the case for the AtxA regulator in *B. anthracis*, or for the CpcR and VipR regulators in Bt strains LM1212 or *kurstaki* HD1 (50, 51). Of course, many other genes potentially involved in adaptive functions are present on these large plasmids, but apart from those responsible for sporulation, aggregation, and conjugation, most of these functions remain unknown (52–54).

The Bt plasmids are also a rich source of mobile genetic elements. The close association between the pesticidal protein genes and transposable elements was initially demonstrated for *cry1A* genes in the *berliner*, *kurstaki,* and *aizawai* Bt strains active against lepidopteran insects. In these strains, the *cry1Ab* gene was shown to be flanked by two series of inverted DNA sequences (55, 56). These DNA sequences have been identified and characterized as IS*231* and IS*232* sharing similarity with the IS*4* and IS*21* families, respectively (57, 58). In the Bt strain *israelensis*, several copies of the insertion sequence IS*240* of the IS*6* family are located on the large plasmid pBtoxis, harboring all the *cry* and *cyt* genes contributing to the activity of this strain against mosquitoes (48, 59). The close association observed between various IS and *cry* genes (55, 56, 58, 60) suggests that these mobile elements and toxin genes form pathogenicity islands and composite transposons.

In addition to insertion sequences, Bt was also the source of a specific replicative transposon belonging to the Tn*3* family but uses a tyrosine recombinase, TnpI, to resolve transposition intermediates, rather than a serine recombinase (61). These transposons (Tn*4430* and Tn*5401*) were found in various Bt strains active against lepidopteran (Tn*4430*) or coleopteran (Tn*5401*) insects (56, 62, 63). Tn*4430* is frequently located in the vicinity of *cry1A* genes and does not encode genes other than those involved in transposition (*tnpA*) and site-specific recombination (*tnpI*), suggesting that its main, if not sole, function is to ensure its own propagation or to mediate the horizontal transfer of plasmids within the *B. cereus* group. This process occurs by a conjugation process (previously referred to as conduction) involving the formation of cointegrate molecules between a conjugative plasmid carrying Tn*4430* and a nonconjugative plasmid (64). Since its characterization, Tn*4430* has been used as a model to decipher the molecular mechanisms underlying replicative transposition and study the complex phenomenon of target immunity, whereby a transposon inserts only once into the same DNA target (65, 66). In terms of application, Tn*4430* has been used to develop a clever method allowing the random insertion of a pentapeptide (i.e., a DNA sequence of 15 bp) into target proteins to analyze their activity and identify essential regions (67, 68). TnpI and its internal resolution sites were used for gene replacement and excision of selective markers such as antibiotic resistance genes (69, 70), as well as to develop an *in vivo* expression technology that led to the identification of *B. cereus* genes specifically expressed during infection of insect larvae (71).

Studies on extrachromosomal elements indicate that transposons or insertion sequences associated with conjugative plasmids may have important adaptive functions in mediating the horizontal transfer of genetic material, particularly toxin genes, within the *B. cereus* group. Notably, it was shown that transfer of conjugative plasmids carrying *cry* genes occurs efficiently in infected insect larvae (72, 73). To conclude this chapter, it is clear that Bt is a remarkable model for highlighting the role of plasmids and mobile genetic elements in bacterial diversity and, consequently, in the adaptation of bacteria to their broad ecological niche.

### Diversity of insecticidal toxins

As mentioned above, the crystal inclusions are composed of ∂-endotoxins corresponding to two types of toxins, the Cry and the Cyt proteins (3, 27). Various combinations of toxins can be found in the crystal depending on the *B. thuringiensis* strain; for example, the commercial strain *kurstaki* HD1 Dipel produces bipyramidal and cuboidal crystals consisting of five Cry toxins active against lepidopteran larvae (49); the strain *israelensis* produces roughly spherical crystals containing four Cry toxins and two Cyt proteins active against Diptera, notably against various species of mosquito larvae (48). By adapting to the ecological niche formed by arthropods, particularly Lepidoptera, Coleoptera, and Diptera, but also certain nematodes, Bt has developed a wide range of toxins that appear to fit specifically to these organisms. A nomenclature was proposed in 1998 to classify the various Cry and Cyt toxins produced by Bt (26). This provided a sound framework for protein classification, but the very large number of pesticidal proteins discovered over the last two decades, along with the introduction of proteins forming crystals but showing low sequence and structural similarity with the canonical insecticidal proteins, led to some inconsistencies. A revised nomenclature, based exclusively on sequence and structure similarity, prevents misclassification and reflects the huge diversity of the pesticidal proteins produced by these bacteria (25, 74). (N. Crickmore, C. Berry, S. Panneerselvam, R. Mishra, T. R. Connor, and B. C. Bonning, Bacterial Pesticidal Protein Resource Center, viewed in August 2025, https://www.bpprc.org). In this new nomenclature, Crickmore and colleagues identified 16 structural classes of pesticidal proteins. However, based on their representation and significance in the Bt species, three major groups can be distinguished: the Cry, Cyt, and Vip toxins.

#### The Cry toxins

During the past decades, several hundred *cry* genes have been isolated and cloned from Bt strains. Today, the totality of the Cry proteins can be distributed into some 75 distinct classes sharing less than 45% identity between them (25, 26). In other terms, proteins that shared at least 45% sequence identity were placed in the same primary group (Cry 1, 2, 3…). Thus, the Cry proteins form one of the largest (if not the largest) protein families found in the microbial world. Analysis of the phylogenetic relationships between the various Cry proteins suggests that their diversity and specificity are the result of mutations and sequence swapping by homologous recombination between their respective domains (75, 76).

In the revised nomenclature, all the Cry toxins form a unique family of three-domain proteins. Indeed, on the basis of the 3D structure, the active fraction of these toxins reveals three distinct domains: a domain consisting of seven antiparallel alpha-helices and two domains composed of antiparallel β-sheets (77–79). As an example of their insecticidal spectrum, the toxins Cry1 and Cry2 are active against Lepidoptera, Cry3 against Coleoptera, and Cry4, Cry10, and Cry11 against Diptera (4). These Cry protoxins have two different lengths: 130–140 kDa for Cry1 and Cry4, and about 70 kDa for Cry3, Cry2, and Cry11. The proteins Cry5 active against nematodes also belong to this group (4).

The Cry toxins are pore-forming proteins whose activity requires binding to specific receptors on the brush border membrane of insect midguts (3, 80). Their mode of action has been extensively studied, and the various stages are now relatively well understood (81–83). Briefly, the first step following the ingestion of a crystal inclusion by susceptible insect larvae is the solubilization of the crystal in the midgut. The protoxins are then processed by the trypsin-like proteases of the insect to 65-kDa active toxins (28). Afterward, a complex multi-step mechanism involves the binding of the activated Cry toxin to specific membrane receptors on the surface of the midgut cells. Overall, the data indicate that the sequential binding of the Cry toxins to receptors, such as cadherin-like proteins, results in the formation of a toxin prepore oligomer able to bind membrane proteins such as aminopeptidase N and alkaline phosphatase (84). The binding of the Cry oligomers causes the formation of pores in the epithelial cells of the insect midgut, resulting in an osmotic shock that kills the cells.

#### The Cyt toxins

The Cyt toxins were first discovered in the crystal inclusions of the Bt strain *israelensis* active against mosquitoes and were then found in all the dipteran-active Bt strains (85, 86). Although these toxins seem to be specifically active against dipteran larvae, *in vitro,* they are cytolytic to various insect cells, and at higher concentrations, they are also cytotoxic to other cells, including erythrocytes (87). The Cyt toxins are 27-kDa proteins that require the presence of a 20-kDa accessory protein for crystallization (88). Structural analysis reveals that Cyt consists of a single domain in which two outer layers of alpha-helix wrap around a mixed beta-sheet (89). This structure is in agreement with the pore-forming properties of the Cyt toxins and suggests that pore formation is not mediated by a specific membrane protein receptor.

The insecticidal activity of protein fractions from the crystal inclusion of Bt subsp. *israelensis* was greater than would have been expected from the addition of the activity of the individual fractions. Mixtures of the Cyt1 and Cry11 proteins and also of Cyt and Cry4 proteins were more toxic than if the proteins were fed individually to the larvae, suggesting a synergism between these proteins (90). It has indeed been shown that the protein Cyt1A synergizes with the Cry11A toxin by binding to the intestinal cells of the mosquito larvae and serving as a receptor (91). A similar synergistic mechanism has been observed between Cyt1A and Cry4B proteins (92).

#### The Vip toxins

In addition to proteins forming a crystal inclusion, several Bt strains active against Lepidoptera produce extracellular insecticidal toxins known as Vip3A (vegetative insecticidal protein) (93). Although designated as vegetative proteins, thereby generating an attractive acronym, recent data indicate that the *vip3A* gene expression occurs during the stationary phase and requires a specific transcriptional activator, VipR (50). Vip3A is then secreted, but the mechanism leading to its export remains to be elucidated. Based on the criteria of the revised nomenclature, Vip3A forms a single class of proteins and is now renamed Vip (25).

The toxin Vip exhibits high toxicity against lepidopteran insects belonging to the Noctuidae family. They are generally produced by Bt strains also producing Cry1A lepidopteran-active toxins. The protoxin Vip is an 88-kDa protein that is activated by trypsin-like proteases in the gut of insect larvae (94). After maturation, the toxic fraction of approximately 62 kDa is able to form pores in the intestinal cells of insect larvae, as well as bind brush border membrane vesicles prepared from insect larval midguts (95, 96). Despite the lack of sequence similarity between Vip and Cry, three-dimensional analysis of Vip proteins revealed some structural homology with Cry toxins, suggesting that the mode of action of these two types of insecticidal toxins may share a number of common features (97, 98).

The insecticidal activity of the exported protein Vip and its association with Cry1 proteins showing related activity spectrum (lepidopteran insects) suggests that the larvicidal activity of the Cry proteins is reinforced by the production of Vip proteins, thus increasing the entomopathogenicity of the Bt cells multiplying in the infected insect larvae; moreover, a synergy was observed between Vip and Cry proteins against a lepidopteran insect (99). While each of these toxins has very specific targets, the combination of different proteins (Cry and Cyt, or Cry and Vip) in certain Bt strains gives these strains a broad activity spectrum, enabling the bacteria to colonize a relatively wide range of insect hosts, albeit generally limited to one order, i.e., Lepidoptera, Coleoptera, or Diptera. As we will see below for Bt plants, these combinations also contribute to counteract the resistance to individual toxins in target insects.

### Regulation of ∂-endotoxin gene expression: from *cry* and *cyt* genes to crystals

In some Bt strains, the crystalline inclusion can represent up to 25% of the bacterium’s dry weight at the end of growth (100). Because of its fundamental interest and strong industrial potential, this remarkable level of production has been subject to numerous studies, leading to several original advances. A non-negligible factor involved in the high production of ∂-endotoxins is certainly the plasmid localization of their genes, which confers them a higher copy number than chromosomal genes. But beyond this consideration, three aspects can be highlighted to explain the levels of production observed: transcriptional, post-transcriptional, and post-translational mechanisms.

#### Transcriptional mechanisms

It is quite obvious that the formation of crystals implies that the production of component proteins takes place in non-dividing cells to avoid their dilution during cell divisions. This is what we observe in Bt, where the crystals appear only in the mother cell compartment of sporulating bacteria (101, 102), or more exactly—as we shall see—during the stationary phase. The production of the ∂-endotoxins in the mother cell suggested a link with sporulation, and indeed, early studies on this subject revealed that transcription of a *cry1* involved two overlapping promoters used sequentially during sporulation (103). Following this work, the Whiteley group showed that transcription of this *cry* gene was directed from these two promoters by RNA polymerases containing sigma factors resembling the sporulation-specific sigma factors SigE and SigK from *Bacillus subtilis* (104–106). A genetic approach then confirmed that transcription of the *cry1* gene depended simultaneously on SigE and SigK, thus explaining its efficiency throughout the sporulation process (107). It was further shown that the transcription of many *cry* and *cyt* genes was dependent on sporulation, and the use of transcriptional fusions with reporter genes proved that the promoters of these genes allowed very high levels of expression (108, 109). However, in lepidopteran-active strains carrying a *vip* gene, it has been shown that, in addition to the SigE- and SigK-dependent expression, an earlier transcription of *cry1A and cry2* genes is activated by VipR at the onset of the stationary phase, independently of sporulation-specific sigma factors (110).

Although sporulation-dependent expression of the *cry* gene appears to predominate in the Bt species, other situations may be encountered. In coleopteran-active strains, *cry3* expression is independent of sporulation sigma factors and is likely transcribed by an RNA polymerase associated with SigA, the primary sigma factor in Bacilli (111–113). However, the transition phase regulator responsible for the activation of *cry3* expression at the onset of the stationary phase has never been characterized. In a Bt *spo0A* mutant strain unable to initiate the sporulation process, the *cry3* gene is expressed and leads to the formation of large crystal inclusions that remain encapsulated in non-viable ghost cells (114).

Another noteworthy situation has been observed in a few rare Bt strains that differentiate into two roughly equivalent subpopulations during the stationary phase: spore formers and crystal producers (115, 116), leading to a division of labor that benefits the whole population even if it ultimately results in the death of the crystal producers. It has been shown that a specific stationary phase activator, CpcR, activates *cry* gene expression in the subpopulation of crystal producers (51). Concomitantly, CpcR activates a gene encoding a phosphatase that dephosphorylates Spo0A-P, thereby reducing the level of sporulation in the crystal-producer cells (117). However, the factors controlling the balance between crystal producers and spore formers at the onset of the stationary phase are not known. This remarkable altruistic behavior observed in a few Bt strains is an example of population heterogeneity that will be developed below in “Pathogenic lifestyle.”

#### Post-transcriptional mechanisms

The stability of mRNA plays a key role in gene expression, and it could be hypothesized that ∂-endotoxins produced in large amounts should be encoded by particularly stable mRNAs. In line with this idea, Glatron and Rapoport (118) demonstrated in the 1970s that the mRNAs encoding the crystal proteins are indeed long-lived mRNAs: 13 min versus 1–2 min for the majority of the prokaryotic mRNAs. The reason for this mRNA stability was first partially explained by showing that the 3′ end of a *cry1A* gene forms a stem-loop structure (the transcriptional terminator) that significantly improves the stability of *cry1A* mRNA, presumably by preventing exonucleolytic degradation from the 3′ end (119). Based on the presence of a strong potential terminator at the 3′ end of all ∂-endotoxin genes, this phenomenon might be generalized. A second factor involved in mRNA stability was discovered by analyzing the *cry3A* gene expression. It was observed that the 5′ extremity of the *cry3A* mRNA did not correspond to the transcription start site but was generated by the presence of a Shine-Dalgarno sequence unable to initiate translation. The presence of this DNA sequence, called STAB-SD, resulted in a 10-fold increase in the downstream mRNA (120). From these data, it was postulated that the binding of a 30S ribosomal subunit to STAB-SD stabilized the *cry3A* mRNA by protecting its 5′ end from 5′-to-3′ nuclease activity (120). However, at that time, no 5′-to-3′ exoribonuclease activity was known in bacteria. Following this result, a team working on mRNA stability confirmed the binding of a 30S ribosomal subunit to STAB-SD and, more importantly, discovered that the essential ribonuclease Rnase J1 was responsible for the 5′-to-3′ decay of mRNAs in prokaryotes (121).

#### Post-translational mechanisms

Whatever the mode of expression of the *cry* or *cyt* genes, the ∂-endotoxins must be produced in a suitable formulation to be delivered into the environment in a concentrated and sufficiently stable form to induce toxemia in an insect larva. Given that spores and ∂-endotoxins are not closely associated at the end of sporulation, after ingestion by susceptible larvae, the effect of the toxins should enable Bt (or *B. cereus*) spores to germinate and cause septicemia. The most effective process adopted in the course of evolution was to produce these toxins in a crystal form, and Bt has evolved two main strategies for achieving this. All Cry proteins of 130–140 kDa (e.g., Cry1 and Cry4 toxins) possess a C-terminal extension, representing approximately the C-terminal half of the protoxin, which is dedicated to crystallization (3). The smaller Cry toxins, such as Cry2 or Cry11, as well as Cyt toxins, require auxiliary proteins to form a crystalline structure. These helper proteins are encoded by genes forming an operon with their respective *cry* or *cyt* genes (88, 122, 123). In addition to these two categories, the Cry3 proteins lack the crystallization domain and do not require helper proteins, suggesting that the intrinsic properties of these proteins are sufficient for the formation of the crystalline inclusion (77).

### Pathogenic lifestyle

The close relationship between Bt and insects is clearly demonstrated, if only by the production of specific insecticidal toxins. But many other factors, such as chitinases, various proteases, siderophores, mechanisms of protection against antibacterial peptides, etc., reinforce the view that insects are indeed the preferred ecological niche of Bt (124, 125). Consequently, choosing an insect to study the infectious cycle of this bacterium was a logical option. Given the knowledge acquired on the genetics of *Drosophila*, this insect could have been the best choice, and trials were conducted with this in mind (126). However, this non-target insect was not suitable as an infection model for assessing the pathogenicity of Bt, as its low susceptibility to these bacteria through oral exposure limited the relevance of the results. For this reason, and for additional considerations detailed below, the larva of the lepidopteran insect *Galleria mellonella* was selected as the infection model.

#### The *G. mellonella* insect model

The *G. mellonella* lepidopteran insect larva infection model is a key experimental system that has enabled numerous advances in understanding virulence, notably in bacteria of the *B. cereus* group. This insect was described as a genetic model in 1938, thanks to its resilience, high fecundity, and ease of laboratory use (127). In recent years, ethical and economic concerns surrounding vertebrate models have driven the adoption of alternatives like *G. mellonella*, especially under the 3Rs principle (Replace, Reduce, Refine). *G. mellonella* rearing is inexpensive relative to mammal models and allows for high-throughput, large-scale experimentation within short timeframes. It is commonly used as an infection model for a broad range of pathogens, particularly in preliminary screening assays and toxicological studies (128, 129). Even though the absence of an adaptive immune response might be a limitation in some studies, the innate immune system of *G. mellonella* shares key features with mammals, making it relevant for modeling infection (124). Furthermore, ease of manipulation allows for force-feeding or intrahemocoelic infections, depending on the pathogen and the question being addressed. The larvae can be incubated across a wide temperature range (4°C–37°C), allowing studies under optimal pathogen growth conditions. Turning now more specifically to the Bt/insect relationship, *G. mellonella* has been used from the 1950s to 1960s to study the virulence of *B. cereus* and Bt, demonstrating the role of phospholipases in virulence (24, 130). Moreover, it appeared that *G. mellonella* was an interesting model for assessing the virulence of the bacterium because the larvae are susceptible to the ingestion of spore-crystal mixtures but poorly susceptible to the crystals alone (131–134). Thus, this insect is a valuable *in vivo* model for studying bacterial pathogenicity and has demonstrated, for example, the important role of iron homeostasis regulation during infection by Bt or *B. cereus* (135, 136).

#### Quorum sensing and phenotypic heterogeneity

Even though Bt gained its fame thanks to its insecticidal proteins capable of inducing mortality in susceptible hosts, this bacterium is also a model for the production and regulation of an arsenal of other factors, shared with members of the *B. cereus* group such as the opportunistic human pathogen *B. cereus sensu stricto* (2, 124, 125). The functions of these factors highlighted the importance of the bacterium itself in the infectious process. This dimension was first revealed by using the synergism between the bacteria and Cry toxins. In the oral infection model of *G. mellonella*, mortality of the larvae was observed solely when spores or vegetative bacteria were force-fed, in addition to the Cry1C toxin to which they are not naturally susceptible (137). This work also showed the fundamental role of the PlcR regulator in the pathogenicity of the bacterium, as a deletion mutant of this transcription factor was no longer able to kill its host. PlcR had been characterized a few years ago as the activator of *plcA*, which encodes a phospholipase (138) and was later demonstrated to control the expression of over 45 genes, most of which encode secreted molecules (139, 140). Infection in *G. mellonella* also allowed determining the pathogenic role of individual-specific Bt or *B. cereus* PlcR-regulated factors, such as a metalloprotease, a collagenase, or a sphingomyelinase (141–143).

**Fig 1:**
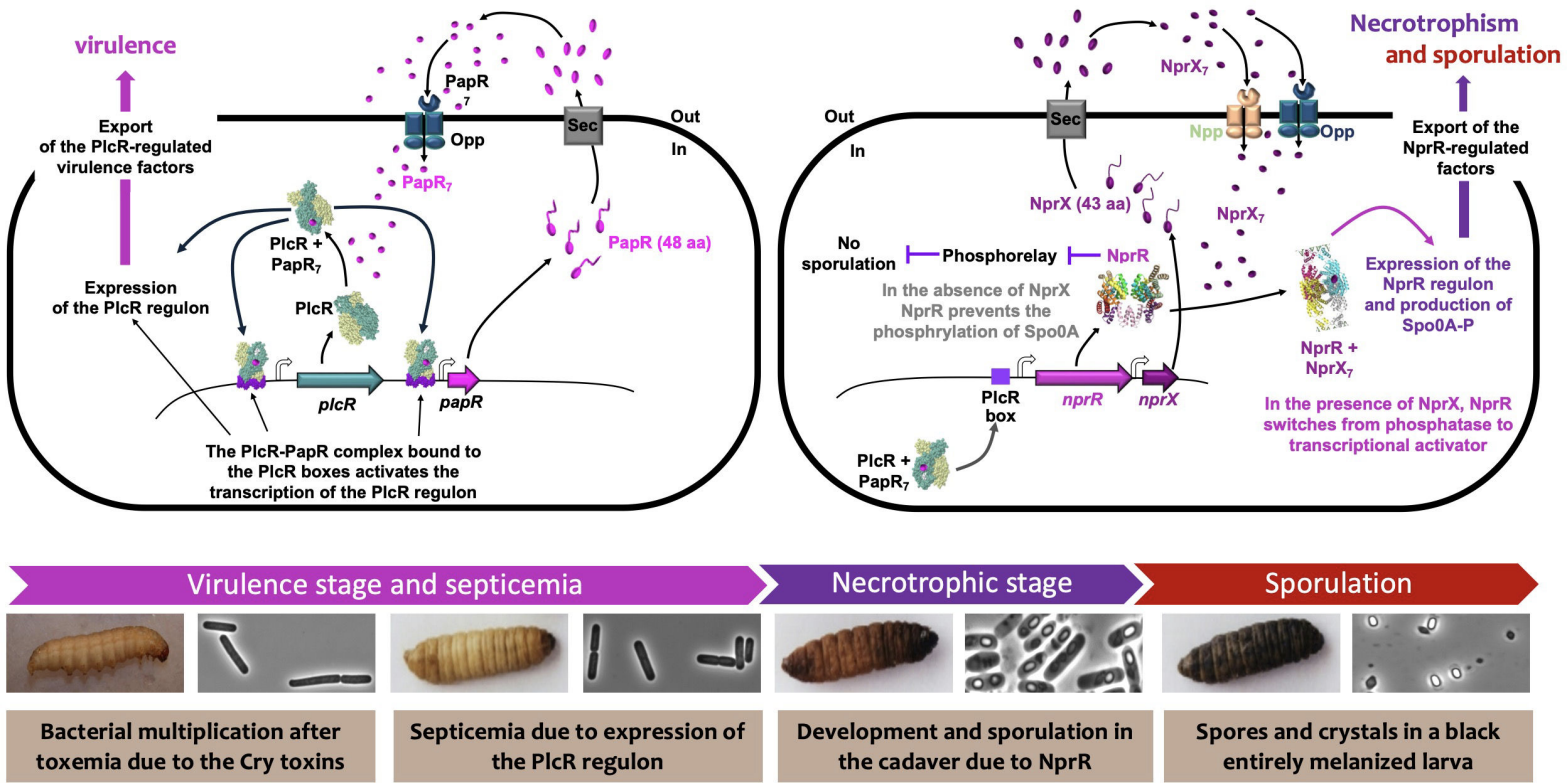
Activation of PlcR-PapR and NprR-NprX quorum-sensing systems during the infection process. From left to right: after toxemia due to Cry toxins, Bt spores ingested together with the crystals germinate and bacteria grow in the midgut of the insect larva (here, *G. mellonella*). The PlcR-PapR QS system is activated at the onset of the stationary phase when the bacterial density is high. At this time, the intracellular concentration of the signaling peptide, PapR, increases. PapR is exported outside the bacterial cell, is matured as a heptapeptide (seven amino acids), is reimported into the bacteria via the oligopeptide permease Opp (150), and induces a conformational change of the regulator PlcR, which is positively autoregulated. The PlcR-PapR complex binds the PlcR boxes and activates the transcription of the PlcR regulon. Virulence factors are produced and exported outside the bacterial cell, causing damage to the intestinal barrier and allowing bacteria to access the hemocoel, inducing septicemia and leading to the insect death. After this stage, which is highly dependent on PlcR and leads to the death and invasion of the infected host, part of the population enters necrotrophism, a state that allows the bacteria to feed on the insect cadaver. This pathway depends on the NprR-NprX QS system that regulates the expression of genes encoding various degradative enzymes. The production of the signaling peptide NprX, its maturation, reimport via two oligopeptide permease systems (Opp and Npp) (151), and the increase in its intracellular concentration induce a conformational change in the NprR regulator, causing it to switch from phosphatase activity to transcription factor activity. As a result, the bacteria are able to exploit the nutrients in the cadaver and, ultimately, sporulate by phosphorylating the Spo0A regulator, the key factor responsible for triggering sporulation. It is notable that sporulation originates in cells that have activated the NprR regulon. At the bottom of the figure, the bacteria are shown from the vegetative growth phase to sporulation and crystal production; the insect larvae are shown alive, dead, partially melanized (brown), and entirely melanized (black). This figure is inspired by those previously published by Slamti and colleagues (152).

The regulation of PlcR-dependent genes was shown to rely on the QS signaling peptide PapR (144), and mechanistic as well as structural studies allowed the understanding of this finely tuned regulation (145, 146) linking QS to the metabolic state of the bacterium (147) (Fig. 1). The PlcR regulon is critical for Bt to cross the gut epithelial barrier after ingestion by the larvae (148), but it was also shown to be important for the pathogenicity of the bacteria following intrahemocoelic injection, indicating that it also plays a role in this compartment of the larvae (149).

Following death and invasion of the larva, part of the population enters necrotrophism (Fig. 1), a state that allows the bacteria to feed on the cadaver (153, 154). This pathway was also shown to depend on QS through the NprR-NprX regulator-peptide pair that regulates the expression of 45 genes (153, 155, 156). In addition to controlling necrotrophism, NprR also moonlights as a phosphatase involved in the repression of sporulation, through binding of Spo0F—a component of the sporulation phosphorelay—before NprX reaches an intracellular level sufficient to induce a conformational change leading NprR to switch to a transcription factor (157–159) (Fig. 1). As a result, the bacteria that enter sporulation originate from cells that have activated the NprR regulon (154). The study of the non-necrotrophic non-sporulated subpopulation later led to the identification of bacteria able to persist in the cadaver of the host for a prolonged period. These cells represent about 50% of the population, are in a slowed-down metabolic state, and present an increased oxidative stress response (160). However, the mechanisms leading to this physiological state remain to be elucidated.

The characterization of the PlcR and NprR regulators, along with pioneering findings on Rap-Phr-mediated QS involved in sporulation and competence in *Bacillus subtilis* (161, 162), led to the definition of the RNPP family of QS regulators that control major functions in gram-positive bacteria, which is now frequently referred to as the RRNPP family (145, 158, 163, 164).

#### Ecology and cheating

We have mentioned above that certain strains can exhibit altruistic behavior thanks to a division of labor that condemns part of the population. However, more generally, the Bt species offers other remarkable examples to study social microbiology, notably because of the production of public goods either via insecticidal crystals that benefit all bacteria that come across them, or via secreted signals and virulence factors through QS systems. Bt naturally colocalizes with other related species that do not produce Cry toxins (165, 166), and QS-null mutants were isolated from Bt and *B. cereus* culture collections (167). These examples raise important questions about the metabolic cost of these systems for the producing cells and the potential benefits they may offer to non-producing cheater strains that exploit public goods without contributing to their production. Cry toxin production in Bt is maintained despite its high metabolic cost through a dynamic balance between cooperative producers and non-producing cheaters. Negative frequency- and density-dependent selection stabilizes cooperation, while soil reservoirs allow periodic re-establishment of clonal populations. These social interactions explain both the persistence of Cry toxins and the rarity of Bt epidemics in insects in natural environments (168–170). Regarding QS, this issue has been specifically investigated for the PlcR-PapR system, where *plcR*- or *papR*-null mutants outcompeted wild-type strains *in vitro* but showed no fitness advantage *in vivo* in a *Plutella xylostella* oral infection model (148). This was likely due to a spatial bottleneck imposed by the host gut, which limits bacterial interactions and prevents cheaters from accessing public goods produced by wild-type cells. These findings highlighted the importance of studying QS in natural ecological contexts, where the host and its spatial structure impose selective pressures not captured in simplified laboratory models.

## FUTURE: FUNDAMENTALS AND APPLICATIONS

### Fundamentals

The single-cell approaches performed in several studies presented above have revealed that Bt does not form a homogeneous population during infection, even though many of the processes involved depend on QS. As mentioned, not all bacteria enter necrotrophism and consequently sporulation, contrary to the virulence pathway. However, the expression of PlcR-regulated genes remains to be studied in the insect gut, which could reveal differences in the activation profile observed when bacteria are injected into the hemolymph (154). Although realized in a natural setting, all previously presented *in vivo* analyses of QS were conducted on bacteria extracted from their host, which precludes the investigation of whether spatial patterns of gene expression are influenced by the host-associated environment. Indeed, the host represents a structurally and microbiologically complex ecosystem potentially capable of modulating gene expression that might lead to the emergence of phenotypically distinct subpopulations. Factors such as nutrients, oxygen, or other molecular gradients—naturally arising within the host—can further contribute to this heterogeneity. For instance, it has been shown that *Vibrio cholerae* cells located near the epithelial surface of the intestine in a rabbit ileal loop model express higher levels of virulence genes compared to those in the intestinal lumen (171). This differential expression was attributed to bicarbonate production by epithelial cells in this region of the gut. Differential gene expression was also observed in *B. cereus* isolated from the gut of *G. mellonella* at a post-infection time point when the bacteria were predominantly in a biofilm-like state, compared to an earlier time point where they appeared primarily as individual cells within the lumen (135). The removal of the bacteria from the host results in the loss of critical spatial information of bacterial communities, such as microcolonies or biofilms. These structural arrangements may play a pivotal role in regulatory processes, notably those governed by QS systems. It is, therefore, important to analyze these pathways *in situ*, minimizing disturbances to the host to preserve the native architecture and dynamics of infection. Bt offers a powerful model for such investigations due to its genetic amenability and the development of tools enabling single-cell resolution analysis coupled to the availability of natural and simple infection models such as *G. mellonella* or *Caenorhabditis elegans* (172, 173).

The multifaceted lifecycle of Bt can be leveraged as a model to explore the evolution of a bacterial infection in a host through *in vivo* real-time imaging using single-cell approaches integrated with advanced microscopy techniques. These approaches will not only provide new insights into Bt pathogenesis but also offer a broader framework for understanding infection processes and differentiation dynamics in other members of the *B. cereus* group and beyond, under conditions that closely mimic the natural host environment. An interesting experimental approach offered by the Bt-insect infection model is the ability to study the role of the intestinal microbiota during infection. In the case of insect larvae intoxicated with spore-crystal preparations of the Bt *kurstaki* Dipel strain, it was observed that bacteria of the indigenous gut microbiota, rather than the Bt cells, played an essential role in the septicemia resulting from the toxemia caused by Cry toxins (174, 175). This conclusion was later challenged by studies suggesting that the results were due to the use of antibiotics inhibiting the growth of Bt in the insect (176, 177). Furthermore, in the specific case of *G. mellonella*, it has been shown that an *Enterococcus*-dominated gut microbiota has a protective effect against infection caused by Bt spores and Cry toxins (178), and that opportunistic human pathogens such as *Enterococcus faecalis* do not synergize with the activity of Cry toxins administered orally to larvae, unlike Bt or *B. cereus* strains (134). The pathogenicity of Bt toward insect larvae is supported by the above-mentioned results, which show that deletion of a regulatory gene (*plcR*) abolished the virulence of Bt when administered orally and, to a lesser extent, after intrahemocoel injection in *G. mellonella* (137, 149). However, using another lepidopteran insect, *Manduca* sexta, it has been shown that *E. faecalis* can induce a septic death after treatment with the Cry1Ac toxin (179). The role of the midgut microbiota in septicemia following Cry toxin treatment was also observed by using RNA interference to disrupt cellular immune barriers in the Lepidoptera *Spodoptera littoralis* (180). It is clear from these controversies that many parameters must be taken into account in this type of study, and the contradictory results observed in these experiments may be due to the insect species used, their diet, their microbiota, or other factors that are difficult to control. Given their potential importance, studies on the role of the intestinal microbiota in bacterial infections need to be further investigated and may benefit from the advantages offered by the manipulation of the Bt-insect couple.

#### Applications

Protein crystallization holds significant industrial potential. In the context of applying the properties of Cry toxins, the C-terminal domains of Cry1 and Cry4 proteins have been utilized to produce heterologous proteins such as antigens and Vip3. In both cases, protein production was enhanced, and inclusion bodies were formed. However, the crystal structure and biological activity of the resulting fusion proteins were not successfully achieved (181, 182). Therefore, the use of crystallization domains or auxiliary proteins alone is insufficient to produce well-ordered crystalline structures without a thorough understanding of the underlying molecular interactions. The crystal inclusions naturally formed by Bt are the result of coevolution between several factors, including the toxic moiety, crystallization domains, and accessory proteins. Elucidating the molecular basis of these interactions is critical for the rational design and successful crystallization of heterologous proteins using these elements.

Regarding biological control, Bt-based biopesticides have enjoyed undeniable success in the fight against insect pests for several decades, thanks to their effectiveness and high specificity. Despite this, their use remains marginal compared with chemical insecticides. To deal with the problems associated with the massive use of chemical insecticides, Bt-based biopesticides certainly need to be further improved to make them easier to use and more attractive to farmers and public authorities. To a lesser extent, these comments also apply to vector control. Even though the Bt *israelensis* strain has led to major successes in this field, its use remains relatively low while the health problems posed by mosquitoes continue to increase throughout the world, particularly as a result of climate change.

Commercial formulations of Bt consist of a mixture of spores and crystals. With the increasing use of these biopesticides in agriculture and vector control, concerns have emerged about their potential adverse effects on non-target organisms, as well as their possible implication in foodborne poisoning (183–185). Although the detection of Bt on various vegetables is expected, given its environmental ubiquity and widespread application, current evidence does not establish a causal link between Bt strains and cases of gastroenteritis. Nevertheless, the increasing use of Bt-based products warrants careful consideration of the potential risks associated with increased exposure. To definitely solve this problem, it is essential to characterize the factors responsible for diarrheal syndromes caused by certain strains of *B. cereus* in order to determine whether commercial strains of Bt do or do not possess such properties. One alternative strategy to address these concerns would be to use sporulation-deficient (Spo⁻) Bt strains that are non-viable in the environment (107, 114, 186). Additionally, Spo⁻ strains retain Cry crystals within the intact cell wall due to the absence of lysis caused by sporulation. This encapsulation confers increased stability to Cry toxins, particularly by enhancing their resistance to UVs, thereby helping to prolong their bioactivity under field conditions (187).

We cannot conclude this history of Bt without mentioning the considerable impact this bacterium has had on agriculture through the use of its toxins in transgenic plants. Since the initial work carried out by the Belgian company Plant Genetic Systems on the transformation of tobacco plants with a *cry1A* gene (188), great progress has been made in this field, and crops genetically modified with Bt genes now account for a significant proportion of the cultivated area in many countries. The use of Bt plants in agriculture has led to in-depth consideration of the problems associated with the emergence of resistant insects. Two main strategies have been implemented: the “High Dose/Refuge” strategy (189) and the “pyramid strategy” (190). The first one involves the use of Bt plants that produce high concentrations of insecticidal toxins to kill all the target pests, in combination with refuge zones where the insects are not subject to the selection pressure exerted by the toxins. The second strategy consists of associating several Bt genes (e.g*., cry* and *vip*) into one plant, encoding toxins that target the same insects but bind to different midgut receptors. This latter strategy reflects that adopted by a number of Bt strains, which contain various *cry* and *vip* genes, as is the case for the commercial Bt strains Dipel and Xentari. This natural evolutionary process enables sustained efficacy by managing insect resistance, as demonstrated for the Bt *israelensis* strain against mosquitoes (191).
